# The effectiveness of pharmacist-led educational model in adult patients with allergic rhinitis: a single-center randomized control trial protocol (AR-PRISE RCT)

**DOI:** 10.1186/s13063-024-08111-y

**Published:** 2024-04-25

**Authors:** Chii-Chii Chew, Xin-Jie Lim, Pathma Letchumanan, Doris George, Philip Rajan, Chee Ping Chong

**Affiliations:** 1 Discipline of Clinical Pharmacy, School of Pharmaceutical Sciences, Universiti Sains Malaysia, Minden, Penang 11800 Malaysia; 2 Clinical Research Centre, Hospital Raja Permaisuri Bainun, Ministry of Health, Level 4, Ambulatory Care Centre (ACC), Jalan Raja Ashman Shah, Ipoh, Perak 30450 Malaysia; 3 Department of Otorhinolaryngology—Head and Neck Surgery, Hospital Raja Permaisuri Bainun, Ministry of Health, Ipoh, Perak Malaysia; 4 Pharmacy Department, Hospital Taiping, Ministry of Health, Taiping, Perak Malaysia

**Keywords:** Rhinitis, Allergic, Pharmacist, Education, Pharmaceutical service

## Abstract

**Background:**

Allergic rhinitis is a chronic respiratory disorder that significantly impacts patients’ quality of life (QoL) and work performance. Pharmacists are recognized as suitable professionals to provide patient education and pharmaceutical care for managing allergic rhinitis patients. However, local clinical practice guidelines, particularly regarding pharmaceutical care in public healthcare institutions, are lacking. This study protocol outlines a randomized controlled trial (RCT) designed to evaluate the effectiveness of a pharmacist-led educational model (AR-PRISE Model) in managing allergic rhinitis in adult patients compared to standard pharmaceutical care. The AR-PRISE model delivers patient educational material and a pharmaceutical care algorithm.

**Method:**

This is a 6-month, single-center, prospective, randomized, two-arm, and parallel-group controlled trial. The trial recruits patients attending the otorhinolaryngology clinics of a tertiary referral hospital. Participants are randomized into control or intervention groups in a 1:1 ratio using permuted block randomization. The total number of participants estimated is 154, with each group requiring 77 participants. The control group receives standard pharmaceutical care, while the intervention group receives pharmacist-led education according to the AR-PRISE model. Both groups are assessed for middle turbinate endoscopy findings, disease severity, knowledge level, symptom control, medication adherence, and QoL at baseline and the end-of-study follow-up (day 180 ± 7). Depending on feasibility, intermediate follow-ups are conducted on days 60 ± 7 and 120 ± 7, either virtually or face-to-face. During intermediate follow-ups, participants are assessed for symptom control, medication adherence, and QoL. The intention-to-treat analysis includes all participants assigned to each group. An independent *T*-test compares the mean difference in knowledge level between the two groups. A two-way repeated measures ANOVA analysis is employed to determine between-group differences for scores of symptom control, adherence rate, and QoL. A *P*-value < 0.05 is considered statistically significant.

**Discussion:**

This study protocol will provide a framework for conducting a randomized controlled trial (RCT) to evaluate the effectiveness of pharmacist-led education intervention in managing allergic rhinitis within public healthcare settings. The parameters measured in this trial will quantify outcomes associated with improvements in symptoms and QoL. By systematically assessing these outcomes, we aim to contribute valuable insights into the role of pharmacist-led interventions in enhancing the management of allergic rhinitis in public healthcare settings.

**Trial registration:**

ClinicalTrials.gov NCT06027736. Registered on 9 July 2023—retrospectively registered.

**Supplementary Information:**

The online version contains supplementary material available at 10.1186/s13063-024-08111-y.

## Introduction

### Background and rationale

In parallel with its increasing global prevalence over the past few decades [1], allergic rhinitis has been classified as a serious chronic respiratory disorder due to its high incidence and negative effects on quality of life (QoL). It is characterized by symptoms such as nasal blockage, sneezing, nasal itchiness, and rhinorrhea following exposure to allergens that trigger immunoglobulin E (IgE)-mediated reactions [2]. In addition to environmental pollution, immunologic interactions with allergenic proteins, such as food and potentially insect bites, can trigger IgE-mediated allergic reactions. These interactions are potential contributors to the increasing incidence of this allergic airway disease [3, 4].

Allergic rhinitis can significantly impair sleep quality and severely affect leisure time, social interactions, academic achievement, and job productivity [2]. This condition also carries significant direct and indirect costs. Examples of indirect costs include sick days, absences from work and school, and lost productivity [5]. Fortunately, widely available treatments can improve outcomes for rhinitis and related allergy illnesses such as asthma. Common pharmacological treatments include oral, intranasal, or ocular H1-antihistamines; intranasal corticosteroids, or a specific combination of intranasal H1-antihistamines and corticosteroids [2]. However, despite these treatment options, allergic rhinitis remains underdiagnosed and undertreated [6].

Recognizing the chronic nature and escalating prevalence of allergic disorders, the World Allergy Organization (WAO) has expressed concerns about their significant impact on healthcare systems and QoL. The WAO White Book on Allergy contains information about the global allergy epidemic and includes a “Declaration of Recommendations” directed at governments and healthcare policymakers [7]. One of the recommendations entails exerting efforts to enhance public awareness and actively engage in the development of innovative prevention strategies [7].

Moreover, patient education has been shown to be effective in increasing compliance among patients with allergic rhinitis [8]. However, patients often express frustration over conflicting and fragmented information delivered by different healthcare providers [9]. In addition to prescription counseling, pharmacists play a crucial role in delivering information on allergic rhinitis management [10, 11]. They can assist patients in setting goals for managing their symptoms, leading to better long-term symptom control and QoL [9, 12, 13]. Despite the crucial role of pharmacists in patient education and counseling, studies evaluating pharmacist-led interventions in allergic rhinitis remain limited. Only four studies were conducted between 2000 and 2020, focusing on educational interventions, mixed-method pharmacist versus patient-goal settings, and a case study observing the impact of pharmaceutical care [12–15]. Further research is needed to determine measurable outcomes in patients with allergic rhinitis [16].

A pharmacist-led educational model that standardizes communication, covering aspects such as the nature of the disease, pharmacotherapy choices, treatment expectations, allergen identification, and intranasal corticosteroid (INCS) administration techniques, is deemed necessary. This model should be structured to facilitate accessible communication with other professionals, such as physicians and nurses. However, the role of pharmacists in the public healthcare setting has been limited in the area of INCS counseling. Typically, pharmacist-conducted counseling has focused mainly on INCS spray demonstrations. The ARIA pharmacy guideline and literature recommend that pharmacists’ counseling could expand to include non-pharmacological management, such as educating patients on allergen identification and exposure avoidance [12, 17, 18].

Some countries operate on a two-tiered healthcare system, comprising public universal healthcare (government-funded) and private healthcare (self-funded or private insurance). Pharmacy practice in government-funded settings differs from community pharmacy practice in private settings [19]. The ARIA guidelines primarily focus on community pharmacy practice and may not be readily adaptable for implementation in government-funded facilities [12, 20, 21]. Strengthening allergic rhinitis management necessitates the development of strategies, tools, and policies [22].

Currently, there is a lack of specific Malaysian Clinical Practice Guidelines for the management of allergic rhinitis. The only local guideline mentioned in relation to allergic rhinitis pertains to the management of rhinosinusitis in adolescents and adults. However, this guideline lacks detailed information, focusing solely on the choice of corticosteroid nasal spray and its administration technique. In the Malaysian context, the Allergic Rhinitis and its Impact on Asthma (ARIA) guidelines are commonly referenced for the management of allergic rhinitis [23–25]. A recently published consensus statement deliberates on recommendations for the pharmacological management of allergic rhinitis in Malaysian patients. The treatment strategies outlined begin with allergen avoidance, followed by oral antihistamines, and escalating care to INCS. Thereafter, a step-up approach may involve the combination of INCS and intranasal antihistamines. Leukotriene receptor antagonists can be considered in cases of co-existing allergic rhinitis and bronchial asthma. Lastly, given the risk of systemic adverse effects, allergen immunotherapy can only be considered when the patient refractory to the optimal treatment of INCS and oral antihistamine as recommended. The consensus statement aims to complement existing guidelines rather than define the standard of care for allergic rhinitis [26].

Our literature search indicates a need for a local pharmacist-led model in public healthcare settings. International guidelines recommend establishing a local standard of care that takes into account pharmacy practice, infrastructure, workforce, and regulation [21].

## Objectives

The objectives of this study are to evaluate the effectiveness of a pharmacist-led educational model in managing adult patients with allergic rhinitis. The parameters used to assess the effectiveness of this intervention include patient knowledge level, symptom control, medication adherence, and quality of life (QoL).

## Trial design

This is a 6-month, single-center, parallel-group, two-arm, exploratory RCT comparing the effectiveness of a pharmacist-led education model intervention to usual care in patients with allergic rhinitis in an otorhinolaryngology clinic of a tertiary referral hospital. Participants diagnosed with allergic rhinitis will be recruited at the clinic. They will then be randomized into control or intervention groups at a 1:1 ratio. The control group will receive standard pharmaceutical care, which involves patients obtaining medication from the outpatient pharmacy following a medical consultation session at the otorhinolaryngology clinic. Meanwhile, the intervention group will receive pharmacist-led education, consisting of delivering educational material and algorithms of pharmaceutical care in allergic rhinitis management.

Baseline data will be collected, and both groups will be followed up post-randomization on day 60 ± 7, day 120 ± 7, and day 180 ± 7. Participants will be contacted for follow-up assessments on day 60 ± 7 and day 120 ± 7. Follow-up may be conducted virtually (via Zoom, Google Meet, WhatsApp call, email, or telephone call) or face-to-face, depending on the feasibility and standard operating procedures of the otorhinolaryngology clinic. The end-of-study follow-up will occur during the routine clinic visit around day 180 ± 7.

**Fig. 1:**
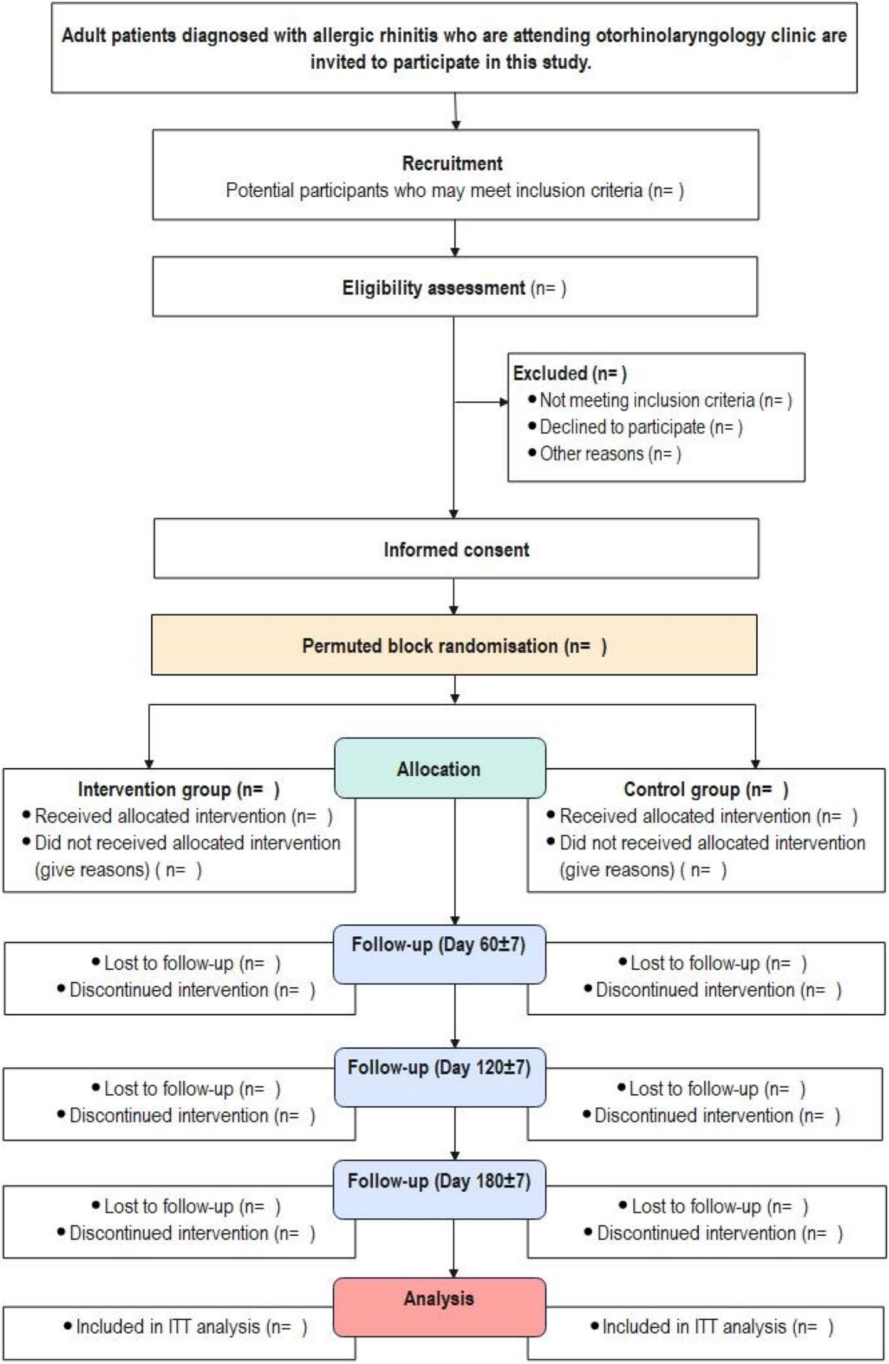
CONSORT flow diagram of the trial design. This study will be conducted and reported on the basis of Consolidated Standards of Reporting Trials (CONSORT) 2010 statement

Patient-reported outcomes, including knowledge level, symptom control, and QoL, will be assessed at baseline using validated questionnaires. Medication adherence will be evaluated through self-recorded INCS administration booklets. Endoscopic features will be determined using middle turbinate grading via endoscopic examination at baseline and at the end of study on day 180 ± 7. During follow-up sessions on day 60 ± 7 and day 120 ± 7, participants will be queried about symptom control and QoL using the same questionnaire, and medication adherence will be assessed. The outcomes of participants randomized to the intervention groups will be compared to those in the control group. A flow diagram of the randomized controlled protocol is illustrated in Fig. 1. This RCT protocol is reported according to the recommendations specified in SPIRIT (Standard Protocol Items: Recommendations for Interventional Trials), and the completed checklist is attached in supplementary material 1.

## Methods: participants, interventions, and outcomes

### Study setting and participants

This study will be conducted at the Otorhinolaryngology Clinic of Hospital Raja Permaisuri Bainun in Perak, Malaysia. This third-largest tertiary hospital in Malaysia is funded by the Malaysian government, provides specialized services and major subspecialties, and serves as the main referral center for the state of Perak. The Otorhinolaryngology Clinic, part of the Otorhinolaryngology Department, offers outpatient services and accepts referrals from primary care clinics and hospitals in the state.

Recruitment will take place at the otorhinolaryngology clinic, where investigators will obtain written informed consent from patients.

### Inclusion and exclusion criteria

This trial will include all adult patients who meet the eligibility criteria during the enrollment period. Inclusion criteria for patients include being aged 18 to 80, Malaysian nationality, diagnosed with allergic rhinitis, capable of reading and writing in English or Malay, and attending the otorhinolaryngology clinic at Hospital Raja Permaisuri Bainun. Participants who received standard care only from this hospital for allergic rhinitis will be included in this study. Patients who are pregnant, are lactating, or have a comorbid diagnosis of chronic rhinosinusitis will be excluded. Female participants who become pregnant after enrolling in the study will be discontinued. Since no investigational product is involved, participants will only be followed up for usual care until the end of the study.

The other exclusion criteria for this study include patients with existing psychiatric problems, dementia, terminal illnesses, comorbidities, and post-COVID-19 conditions with symptoms that persist beyond 3 months after being infected [20], at the discretion of the physician, participants deemed unfit to participate in this study will be excluded. If a participant develops a newly diagnosed psychiatric problem during their participation in this study, they will be excluded from the study and will instead receive their usual care with the Otorhinolaryngology Department at Hospital Raja Permaisuri Bainun.

Post-COVID-19 condition is defined as the continuation or emergence of new symptoms 3 months after the original SARS-CoV-2 infection, including exhaustion, shortness of breath, and cognitive dysfunction, lasting at least 2 months without an alternative explanation [27]. The presence of post-COVID-19 symptoms is assessed and determined by the physician. Allergic rhinitis is categorized as a chronic respiratory condition. Chronic respiratory illnesses have been identified as risk factors associated with an increased incidence of post-COVID-19 symptoms [28]. Therefore, patients presenting with post-COVID-19 symptoms will be excluded from this study.

### Interventions

#### Experimental group

The concept of the pharmacist-led education intervention, known as the AR-PRISE model, is described in a published article [29]. The AR-PRISE model is a locally structured pharmaceutical care led by pharmacists in public healthcare facilities. This model incorporates a patient education protocol and an algorithm of pharmaceutical care in managing allergic rhinitis. The model can be described in two sections as follows: 

##### Section A: Patient educational material

The patient education section consists of 10 topics: (1) background; (2) symptoms; (3) diagnosis; (4) allergic identification and avoidance; (5) INCS consists of general information, priming, administration techniques, cleaning, and addressing concerns; (6) antihistamine; (7) decongestant; (8) alkaline nasal douche; (9) what to do when symptoms flare; and (10) consequences of poor disease control.

##### Section B: Algorithm of pharmaceutical care

Meanwhile, the pharmaceutical care section consists of (1) patient selection, (2) symptom control and assessment, (3) assessing the patient’s QoL, (4) setting a goal of treatment, (5) new user to INCS, (6) existing user, (7) both new and existing users of INCS, (8) teaching patient alert signs, (9) follow-up care, and (10) discharge from pharmacist’s follow-up. The protocol includes a flow chart of the pharmaceutical care algorithm.

A stepwise treatment approach from ARIA guidelines was also adopted to enhance pharmacists’ knowledge [11]. The medications and dosages recommended for treating allergic rhinitis are adapted from the Malaysian Ministry of Health Drug Formulary [30]. A list of relevant medications, categorized by drug category, is included in this section as an appendix to provide quick access for pharmacists.

The model was developed and validated using the modified Delphi technique. Recognizing the heavy workload of pharmacists in public services, the section on patient education was transformed into an audio-visual format—an 8-min video of patient educational material—to facilitate the delivery of patient education. The video underwent testing for its “understandability” and “actionability” before its implementation. The development of the model and the testing of the video on end-users are described elsewhere [31].

The intervention involves the implementation of the pharmacist-led education model. During the intervention, participants will first watch the video and will be given the opportunity to pause the video to ask questions at any time. Following the video presentation, the pharmacist will conduct pharmaceutical care according to the model. Emphasis on the importance of adherence to the intervention will be provided during follow-up visits on days 60 ± 7 and 120 ± 7.

#### Control group

Throughout the trial, the control group will not receive any interventions or instructions to watch videos. Instead, they will receive standard allergic rhinitis management care in an otolaryngology outpatient clinic. During their scheduled appointment date, patients in the control group will have a medical consultation with a physician at the otorhinolaryngology outpatient clinic. Subsequently, they will receive standard pharmaceutical care, which may include advice on drug administration and adherence, when they visit the outpatient pharmacy to fill their prescriptions.

#### Criteria for discontinuing or modifying allocated interventions

The intervention or follow-up is discontinued if any of the following occurs:

1. Participant withdraws consent.
2. The entire clinical trial is discontinued.
3. Non-eligibility is confirmed after registration.
4. The principal investigator or project administrator determines the need for discontinuation.

### Outcomes

The primary outcomes will be the differences in knowledge level, symptom control, medication adherence, and QoL between the control and intervention groups measured from baseline (day 0) to the end of the trial on day 180 ± 7. Differences in mean scores related to knowledge about INCS will be determined by comparing the mean score difference in knowledge level between the two groups. Symptom control between the two groups is determined by the mean score differences. Medication adherence will be estimated through differences in adherence rates, calculated based on the mean number of doses of INCS administered between the control and intervention groups. QoL between the two groups will be evaluated by comparing differences in mean scores on the EQ-5D-5L and EQ-5D-VAS scales.

### Participant timeline

Each participant is estimated to require 6 months for the entire course of participation. The initial encounter is estimated to take 20 min for the interventional group and approximately 10 min for the control group. Follow-up visits on day 60 ± 7 and day 120 ± 7 are estimated to take around 10 min each. The time required for participants at the end-of-study follow-up (day 180 ± 7) is estimated to be similar to the baseline encounter.

### Sample size

#### Knowledge level

Based on the parameters provided and using the G-power calculator with a power of 0.8 and a type I error probability of 0.05, the study requires 70 participants in each group to determine the mean score difference in knowledge level. This calculation is based on the mean score of adult patients with adequate knowledge of allergic rhinitis treatment being 1.39, while the mean score of interventional participants is 1.70 (with a standard deviation of 0.58) [32].

#### Symptom control

The sample size is calculated based on the mean difference in symptom control between the intervention and control groups. An RCT that investigated the change in Total Nasal Symptom Score (TNSS) after an intervention vs placebo in adults in China with intermittent or persistent allergic rhinitis reported a mean score of 3.991 (SD = 2.2145) vs 5.704 (SD = 2.2341) among 181 and 182 participants, respectively [33]. The pooled standard deviation was calculated as 2.2243, and an effect size of 0.3851 was determined. The G-power sample size calculator is used by setting the power at 0.8, the type I error probability at 0.05, two groups, four measurements, a correlation between repeated measurement at 0.5, and the function of repeated measures between factors ANOVA analysis. A sample of 18 participants is required for each group.

#### Medication adherence

A previous study suggested that the mean adherence rate to INCS among the control is 76.62 (SD = 2.85), while the intervention group is 93.94 (SD = 2.84) among 16 participants in each group [34]. The pooled standard deviation and effect size calculated were 2.84 and 3.0290, respectively. Using the G-power calculator, setting a probability (power) of 0.8, the type I error probability at 0.05, and we will need 4 participants in each group.

#### QoL

A study mapping the naso-ocular symptom scores to the EuroQoL 5-Dimensions, 5-Levels (EQ-5D-5L) utility values was referred to estimate the sample size required to compare patients’ QoL between two groups. The EQ-5D-5L utility values in mean and standard deviation are corresponding to the disease severity in allergic rhinitis patients, where the utility values of 1.000 ± 0.000, 0.943 ± 0.085, 0.909 ± 0.095, 0.849 0.142, and 0.767 ± 0.175 corresponding to none, mild, moderate, severe, and most severe groups were referred [35]. In this study, the intervention aims to improve the symptoms, if not totally absence of symptoms, at least the mild disease state. Assuming that the control group patients would be moderate to severe and the intervention group would achieve mild disease status, the EQ-5D-5L utility values corresponding to moderate (0.909 ± 0.095) and mild (0.943 ± 0.085) were selected to simulate the mean utility score of control and intervention, respectively. The pooled standard deviation and effect size calculated were 0.09014 and 0.1886, respectively. The G-Power sample size calculator is used, and by setting the function of a repeated-measure ANOVA statistical test, the probability (power) at 0.8, the type I error probability at 0.05, two groups, four measurements, a correlation between repeated measurement at 0.5, and the sample size of 70 calculated for each group are needed.

The sample size estimation required for EQ-5D VAS was based on a similar study that evaluated general health-related QoL among allergic rhinitis patients. The mean and standard deviation score of EQ-5D VAS of the control group was 72.1 ± 19.0 in 150 patients [36]. The scores of EQ-5D VAS for the intervention group were derived from a study assessing QoL in the general Malaysian population with a reported mean and standard deviation of 85.5 ± 12.3 among 1137 participants, with the rationale that the intervention group would achieve QoL similar to the general population [37]. The pooled standard deviation and effect size calculated were 13.2517 and 0.3245, respectively. Using the G power calculator and setting the function of a repeated-measures ANOVA statistical test, the probability (power) at 0.8, the type I error probability at 0.05, two groups, four measurements, a correlation between repeated measurement at 0.5, and the sample size 25 are calculated for each group.

#### Final sample size estimation

Given the highest sample size requirement calculated for each specific objective, which is 70 participants for each group, and considering a predicted dropout rate of 10%, it is prudent to recruit 77 participants for each group. Therefore, a total of 154 participants will be recruited for this study to ensure adequate power and account for potential dropouts.

## Randomization

Potential participants will be identified during their visits to the otorhinolaryngology outpatient clinic for an evaluation of their condition. Qualified medical doctors who have diagnosed adult patients with allergic rhinitis will refer eligible individuals to the investigators for further assessment and potential enrollment in the study.

### Sequence generation

The participants are allocated through permuted block randomization using randomly selected block sizes. A web-based randomization tool called “Sealed Envelope” is utilized by the assigned research assistant to generate the sequence. The generator accepts block sizes of 4, 6, and 8, with the target sample size set to *n* = 154. Subsequently, a Microsoft Excel file is created containing a list of block identifiers, block sizes, block sequences, group assignments, and a seed number (Seed: 103940014716468) [38]. This list is then managed by the designated research assistant, who is not involved in patient treatment or recruitment.

### Concealment mechanism

The primary investigators and site investigators who obtained written informed consent will be concealed from the randomized number. They will not be involved in the participant allocation process. Instead, they will contact central methods by phone once the patient is consented. The allocation of participants will be performed independently by a designated research assistant.

### Blinding

Due to the nature of the intervention, it is infeasible to mask the participants and the principal investigator, who is the sole person performing the pharmacist-led intervention for the participants in the interventional arm. Both the interventional and control groups received standard care from the study site. Only the intervention group will be receiving the educational protocol (AR-PRISE model), which is deemed low risk to the patients. In the event of any emergencies, blinding will not affect the safety of the patients. The intervention approach did not involve any trial of new investigational drug products or devices. Therefore, there are no plans to allow unblinding. Nonetheless, the otolaryngologists, medical doctors, nurses, and pharmacists who provide usual care will be blinded to the assignment of participants to intervention or control groups.

### Data collection and management

#### Plans for assessment and collection of outcomes

**Fig. 2:**
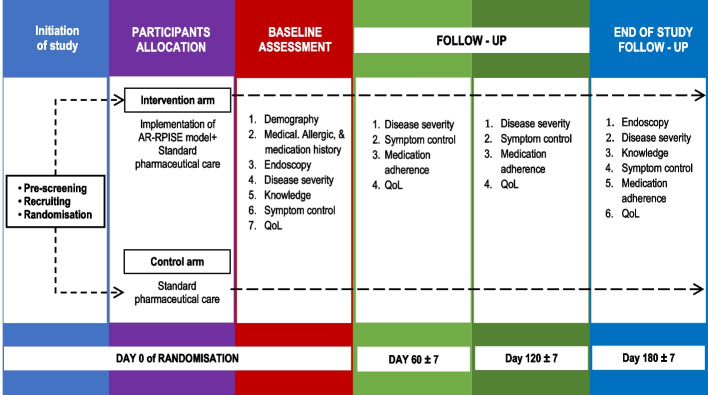
The process of assessment and data collection at different time points

The overview of the assessment and follow-up data collection is illustrated in Fig. 2.

The procedures for conducting this trial at each specific time point range from eligibility screening to the time of intervention to assessments, as shown in Table 1.

**Table 1:**
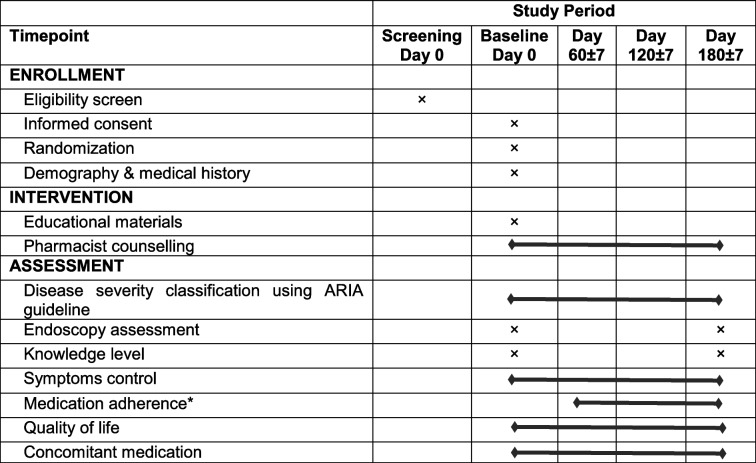
The study schedule of enrolment, interventions, and assessments

#### Demography and assessment form

Participants will provide demographic information including age, gender, occupation, ethnicity, and smoking status upon enrollment. Smoking status will be categorized as “current,” “former,” or “never smoker.” Medical and allergic histories will be assessed, and underlying diseases will be extracted from participants’ medical records.

Subjective severity measurements will be graded using visual analog scales (VAS) at baseline and days 180 ± 7, and ARIA guidelines at baseline and during follow-up visits on days 60 ± 7, 120 ± 7, and 180 ± 7. Physicians will assess the endoscopic findings for middle turbinate edema at baseline and at the end of the study.

Concomitant medications, if any, will be documented. This includes all prescribed and over-the-counter medications taken by the patient in the last 30 days. Details such as medication dose, route, unit, frequency of administration, indication, and dates of administration will be recorded. Concomitant medications of specific interest for the study period include antihistamines, corticosteroids, anticholinergics, leukotriene receptor antagonists, nasal saline, decongestants, and beta-blockers. This information will be captured in the demography and assessment form.

Traditional medicine and supplements taken by the participants for their allergic rhinitis condition will be assessed. Study-specific medications, such as antihistamines, corticosteroids, anticholinergics, leukotriene receptor antagonists, nasal saline, decongestants, and beta-blockers that are prescribed by the physician from the otolaryngology department will be extracted from the hospital information system. This information will be recorded in the demography and assessment form.

#### Measurement knowledge of INCS

A self-administered questionnaire consisting of four items will assess participants’ understanding of INCS [32]. Participants will mark “yes,” “no,” or “not sure” on each item, and their knowledge level will be assessed during baseline data collection and day 180 ± 7.

#### Measurement of symptom control

Total Nasal Symptom Score (TNSS), a validated tool, enables patients to self-rate specific nasal symptoms, including nasal obstruction, itching, sneezing, secretion, runny nose, and sleep difficulty on a 4-point Likert scale ranging from 0 (no symptom) to 3 (severe symptom) in the past 12 h and 2 weeks [39]. Symptom control will be assessed at baseline, on day 60 ± 7, day 120 ± 7, and day 180 ± 7.

#### Measurement of medication adherence

Participants will receive a self-developed diary booklet to record their daily use of INCS. The diary consists of a printed calendar and is designed in pocket size. Participants are instructed to mark the date in the diary for each day they use INCS. Self-recording daily usage of INCS is deemed an appropriate measurement of medication adherence level as it allows a more precise estimation of the number of days the participant declares they are taking the medication. The participants’ adherence level will be assessed on day 60 ± 7, day 120 ± 7, and day 180 ± 7.

#### Measurement of QoL

The EQ-5D-5L, adapted for this study, will be used in allergic rhinitis-related studies [35, 40]. The EQ-5D-5L consists of a descriptive system and the EQ VAS. The descriptive system includes five dimensions (Mobility, Self-Care, Usual Activities, Pain/Discomfort, and Anxiety/Depression), with each dimension consisting of five response levels. Respondents indicate their health by marking the appropriate box for each question. The EQ VAS measures a patient’s overall health perception on a vertical visual analog scale labeled “The best health you can imagine” and “The worst health you can imagine” [37, 41, 42]. The QoL will be measured at baseline, on day 60 ± 7, day 120 ± 7, and day 180 ± 7.

### Recruitment

The recruitment strategies for achieving adequate participant enrollment to reach the target sample size include the researcher actively conducting pre-screening on patients’ clinic cards to identify potential candidates. Eligible patients will be approached on a one-on-one basis in the clinic. Additionally, a staff nurse responsible for the administrative aspects of research in the clinic is designated as a “person of trust.” This individual builds a connection between patients and the project team, facilitating the identification of eligible participants.

### Plans to promote participant retention and complete follow-up

Similar to a previous study [43], this trial will utilize telephone calls or emails to maintain participant retention. Given that follow-up sessions will be conducted virtually during the second and fourth months post-recruitment, they will serve as reminders for participants to remain engaged in the study. Non-responders will receive a maximum of three phone calls per day for three consecutive days. If contact is not established after these attempts, participants will be sent an email containing a questionnaire to solicit their feedback. Should there be no response to the email, a letter will be sent to the participant’s last known mailing address before considering them lost to follow-up.

### Data management

The principal investigator will be responsible for ensuring that the participants’ anonymity is maintained and that the confidentiality of records and documents that could identify participants will be protected, respecting the privacy and confidentiality rules in accordance with applicable regulatory requirements. Individuals involved in this study for medical care, qualified monitors and auditors, and governmental or regulatory authorities may inspect and copy the medical records where appropriate and necessary. All participants will not be given access to their personal information and study data.

Paper case report forms are the primary data collection tool for the study. Site research personnel will subsequently transfer all data into SPSS version 20.0, and the file will be password-protected. The electronic database only allows assigned investigators to enter and view data.

### Confidentiality

Signed informed consent forms will be securely stored in a locked cabinet, accessible only to investigators on site. Participants will be identified solely by their assigned participant identification numbers in all clinical report forms, study-related records, and documents. Additionally, there will be a Subject Identification List containing information such as name, contact number, address, and hospital registration number, along with the corresponding study identification number. This Subject Identification List will be kept in a locked cabinet by the principal investigator on site.

All information obtained during this study will be treated confidentially and handled in accordance with applicable laws and regulations. The participants’ identities will not be disclosed without their consent when publishing or presenting the study results. Investigators involved in the study, qualified monitors and auditors, and governmental or regulatory authorities may access the participants’ medical records when appropriate and necessary.

### Plans for collection, laboratory evaluation, and storage of biological specimens for genetic or molecular analysis in this trial/future use

This study does not involve the collection of biological specimens.

### Statistical methods

#### Statistical methods for primary and secondary outcomes

An intention-to-treat analysis will include all participants assigned to each group after randomization. Data will be analyzed using SPSS version 20.0. The study will evaluate participants’ knowledge levels using a scoring system, where “yes” responses receive a score of two, “no” responses receive a score of zero, and “not sure” responses receive a score of one [32]. The results will be presented as the mean scores and standard deviations for each group. Knowledge levels will be assessed at baseline and on day 180 ± 7. Between-group comparisons will be conducted using *T*-tests, with a *p*-value of < 0.05 considered statistically significant.

TNSS is a 4-point (0–3) scale measuring nasal congestion, sneezing, nasal itching, and rhinorrhea in the past 12 h and 2 weeks. A higher score indicates more severe symptoms: 0 for no symptoms, 1 for mild symptoms easily tolerated, 2 for bothersome but tolerable symptoms, and 3 for severe symptoms interfering with daily activities. The main outcome will be calculated as the mean and standard deviation for each time point. A two-way repeated-measures ANOVA analysis will be used to determine the overall effectiveness of the intervention, with a *p*-value of < 0.05 considered statistically significant.

A two-way repeated measures ANOVA analysis will compare between-group differences in mean adherence rate for INCS doses, calculated as the number of doses taken divided by the total prescribed doses. A *p*-value of less than 0.05 is considered statistically significant.

Participants’ QoL will be analyzed using EQ-5D-5L utility index scores and EQ-5D VAS. Index scores will be generated using Malaysia’s EQ-5D-5L calculator [37]. A two-way repeated measure ANOVA analysis will determine the mean difference between control and interventional groups.

#### Interim analyses

This study does not plan for interim analysis.

#### Methods for additional analyses (e.g., subgroup analyses)

This study does not plan for subgroup analyses.

#### Methods in analysis to handle protocol non-adherence and any statistical methods to handle missing data

No data imputation will be performed for missing data in the primary and secondary endpoints. However, the amount and pattern of missing data in the baseline and endpoint variables will be reported. Potential reasons for any missingness will be investigated and recorded to the greatest extent feasible. Any missing data will be handled in accordance with the conjectured missingness mechanism and the amount of missingness.

### Monitoring

#### Data monitoring

This trial poses a low risk as the intervention involves an educational approach, and both the control and interventional groups are receiving standard care from the study site. Therefore, the formation of a data monitoring committee is not planned for this clinical trial.

However, the monitoring of the entire study trial will be conducted through several steps. Firstly, the principal investigators will provide on-site training on the study method and data collection procedure to all study personnel, including the co-investigators. Procedures for storing the paper case report forms and signed informed consent will be established. Additionally, there will be ongoing remote monitoring on a monthly basis for enrollment rate and protocol adherence by the study supervisor (C-P Chong), who is not involved in recruitment and data collection.

#### Harm

Although this study does not involve new drug investigational products, invasive procedures, or new devices, no foreseeable adverse events are anticipated related to the implementation of this pharmacist-led educational protocol. In the event that participants in the intervention group experience any side effects from prescribed medication, it is unlikely that these would be attributable to the interventional approach introduced to them. Nevertheless, any medication side effects experienced by participants will be meticulously recorded in the assessment form, and patients will be referred to medical doctors for further management.

If participants in either group experience intercurrent illness or observe worsening symptoms, they are advised to promptly seek medical attention at the otorhinolaryngology clinic in this hospital, the emergency and trauma department, or the nearest primary health clinic, without waiting for their scheduled clinic appointment. Participants will also be instructed to contact the principal investigator after visiting any healthcare institution during unscheduled visits. During these visits, participants will be evaluated based on the date of their visit, the healthcare facility they attended, the medication prescribed, and the reason for their visit.

### Ethics and dissemination

#### Plans to give access to the full protocol, participant-level data, and statistical code

The Medical Research and Ethics Committee, Ministry of Health Malaysia, has approved this research, specifying that all records and data are to be kept strictly confidential and can only be used for this study. All precautions are to be taken to maintain data confidentiality. To ensure the data collected is kept confidential, it cannot be shared publicly. However, with a reasonable request, should any party require the data, they can send their request to the principal investigator (chiichii.crcperak@gmail.com), with permission from the Malaysian Director General of Health, before it is shared with any party.

#### Plans for communicating important protocol amendments to relevant parties (e.g., trial participants, ethical committees)

The protocol amendment request to the Malaysian Medical Research Ethical Committee, Ministry of Health, will be made via the website Malaysian National Medical Research Registry.

#### Consent

The investigators include C-C Chew, X-J Lim, P. Letchumanan, and P. Rajan, who are also listed as contributors in this study, have Malaysian Good Clinical Practice certificates, and are involved in informed consent taking. Two otorhinolaryngologists (Dr. Kelvinder Singh Awtar Singh and Dr. Loong Siow Ping) who participated after this study protocol was developed are involved in informed consent taking.

#### Dissemination plans

The study team will report the study findings to stakeholders, including the head of the rhinology service and the pharmaceutical service division. If the pharmacist-led intervention proves effective, the protocol can be shared with the outpatient pharmacy in all government-funded facilities by introducing it to outpatient pharmacists via a workshop [29]. The principal investigator will determine authorship eligibility based on the ICJME guidelines upon the completion of this trial. There is no intention to use professional writers for writing up the completed project.

## Discussion

This study protocol aims to evaluate the effectiveness of a pharmacist-led educational intervention for patients with allergic rhinitis at government-funded healthcare facilities. We referred to a similar RCT protocol, which involved a pilot cluster study with post-intervention follow-up at 1 and 6 weeks [43]. An older study conducted a parallel-group study comparing pharmacist-led interventions to patient goal-set outcomes over 6 weeks [14]. In contrast, our study will be a full-scale RCT with a 6-month follow-up period, allowing us to observe the long-term effects of the intervention. We consider 6 months as long-term, as indicated by a previous study which mentioned a 6-month study on the efficacy of beclomethasone dipropionate nasal spray in allergic rhinitis patients [44].

The conduct of this pharmacist-led education intervention in allergic rhinitis management can contribute data to longitudinal research studies. The outcomes monitoring initiatives could assist researchers to better understand the long-term impact of allergic rhinitis management strategies. The collection of real-world data on patient-reported outcomes over extended periods, this trial can inform evidence-based practice guidelines and quality improvement efforts.

The expected long-term benefits of the pharmacist-led education intervention are anticipated to include a decrease in the need for symptomatic relief medication, fewer flare-ups that necessitate clinic visits, and an improvement in patient’s QoL. Studies of pharmacists specifically trained in allergic rhinitis management have shown significant improvement in patients’ symptom control and QoL compared to pharmacists providing usual care. However, these interventions focused on pharmacists, with patients recruited for outcome assessments [13, 45]. In contrast, our trial focuses on developing a pharmacist-led education model that enables the delivery of standardized education and structured pharmaceutical care. Furthermore, while previous similar studies employed RCTs as the study design, the number of participants was relatively small (*n* = 63 patients) in one study [45] and one was a pilot study (*n* = 60 patients) [13]. In this trial, we calculated the sample size to ensure adequate power in addressing the study outcomes.

Previous studies indicate that the prevalence of allergic rhinitis in Malaysia ranged from 7.1 to 8.7% between 2011 and 2018 [25, 46]. A more recent study covering the years 2017 to 2022 showed a higher trend of allergic rhinitis, with rates between 8.14 and 9.23% [47]. While specific information about expenditures related to allergic rhinitis in Malaysia is lacking, a near 10% disease burden could incur considerable direct and indirect healthcare costs. Asian patients suffering from persistent allergic rhinitis and urticaria experience symptoms for up to 298 days per year, with each patient requiring an estimated annual expenditure of 1137 to 2195 USD [48]. The direct cost of allergic rhinitis management in Turkey was 79 USD per patient, 224 million USD in Korea with a prevalence of 8.4%, and 215 USD per patient per year in India. The indirect cost attributed to absenteeism and presenteeism in Asian countries, including Korea, was reported at 49 million USD, and in India, 460 USD per patient per year [49]. With the implementation of this intervention, self-efficacy in disease management could be improved. The direct benefits for patients are associated with better symptom control, improvement in QoL, and fewer clinic visits. The long-term benefit could be viewed from the prospect of lowering direct healthcare expenditures, and indirect costs such as absence from work could be minimized.

The addition of nasal endoscopic features determination, specifically the middle turbinate edema endoscope grading, sets this study apart from other published trials. This feature enables examination of the appearance of edema at the middle turbinate and is suggested to be a potential specific diagnostic tool for distinguishing between allergic and non-allergic rhinitis. It is an emerging tool proposed for use as a marker of inhalant allergy due to its simplicity. The middle turbinate edema grading system comprises five grades ranging from normal, focal, multifocal, diffuse, and polypoid. The grading scale has excellent positive predictive value (PPV = 85.1 to 91.7%) for all grades except focal edema (PPV = 65.5%) and high specificity (94.7%) [50]. The features of the middle turbinate allow for more precise detection of the study population, monitoring of disease progression, and decision for medical treatment or surgical intervention. In contrast to previous similar studies, the lack of endoscopic features for disease progression and often limited patient-reported outcomes is addressed in this study [13, 16, 43].

The EQ-5D-5L has been chosen to assess participants’ QoL in the study. The decision not to utilize a disease-specific tool, such as the mini-Rhinoconjunctivitis Quality of Life Questionnaire, for evaluating QoL in allergic rhinitis participants is primarily due to the absence of cross-cultural translations of these tools in the Malaysian population. In contrast, the EQ-5D-5L has a validated translated version in the Malay language, locally validated [37]. Furthermore, it aligns well with the naso-ocular symptom severity experienced by allergic rhinitis patients and is considered valid and suitable for adaptation in this study [35].

Patient-reported outcome tools for assessing symptom control are common in allergic rhinitis assessment. The decision to select TNSS is based on expert opinion from otorhinolaryngologists and its simplicity for patient administration. TNSS has been reported to provide an objective measurement of a patient’s self-reported symptoms and has the potential for use in daily clinical practice. It has been widely utilized in RCTs and adopted in many countries. Additionally, TNSS is the most accepted primary efficacy variable for drug approval in allergic rhinitis by the US Food & Drug Administration [51]. Moreover, permission to conduct linguistic validation from English to Malay Language for TNSS has been obtained from the originator, and the translated version is deemed appropriate for local use.

The assessment of participants’ adherence to INCS in this study involves the use of a daily booklet to record their medication intake. While many medication adherence assessment tools utilize questionnaires or scales that require participants to recall their medication intake over a certain period, such methods have inherent disadvantages. These disadvantages include participants’ recall bias, difficulty in responding to questions, and underreporting of non-adherence. Undoubtedly, the daily recording of INCS administration may not be the optimal tool for adherence assessment due to potential response bias. However, this measure enables the researcher, albeit not with 100% accuracy, to evaluate the number of doses participants took over a given period [52].

### Implication

Should the pharmacist-led model be proven to be effective in improving knowledge, symptom control, medication adherence, and QoL in allergic rhinitis patients, the AR-PRISE model could potentially be implemented in clinical practice, yielding several positive impacts:

- Encourage ongoing education and support from pharmacists: Patients can acquire skills in self-managing their symptom control with support from pharmacists. By recognizing triggering allergens and taking preventive measures, patients can make informed decisions for treatments when symptoms flare, leading to better disease control.
- Reduced episodes of symptom exacerbation: Patients with knowledge about allergic rhinitis and the importance of medication adherence could experience fewer episodes of exacerbation.
- Patient empowerment: Patients could feel supported and empowered following education and counseling from pharmacists. They would be aware of the expected outcomes from treatment, feel more confident, and be able to take an active role in managing their disease.
- Preventing disease progression: Better symptom control could prevent the progression of the disease and the development of complications. This, in turn, would reduce the burden on the otorhinolaryngology outpatient clinic, as well as alleviate the burden on the public healthcare setting.

## Trial status

We commenced data collection on June 01, 2023, in adherence to the protocol dated June 30, 2022, Version 1.0. As of now, the study team has successfully enrolled 70 participants. The investigators responsible for obtaining informed consent are currently undergoing training, and we are consistently meeting our weekly recruitment targets. We project to complete participant recruitment by November 2023, with the end-of-study follow-up expected to conclude by May 2024.

### Supplementary Information

**Supplementary Material 1.**

## Data Availability

Not applicable.

## Abbreviations

RCT: Randomized controlled trial
INCS: Intranasal corticosteroid
TNSS: Total Nasal Symptoms Scores
QoL: Quality of life
